# Differential effects of motor cortical excitability and plasticity in young and old individuals: a Transcranial Magnetic Stimulation (TMS) study

**DOI:** 10.3389/fnagi.2014.00111

**Published:** 2014-06-10

**Authors:** Shahid Bashir, Jennifer M. Perez, Jared C. Horvath, Cleofe Pena-Gomez, Marine Vernet, Anuhya Capia, Miguel Alonso-Alonso, Alvaro Pascual-Leone

**Affiliations:** ^1^Department of Neurology, Berenson-Allen Center for Noninvasive Brain Stimulation, Beth Israel Deaconess Medical Center, Harvard Medical SchoolBoston, MA, USA; ^2^Faculty of Medicine, Department of Physiology, Autism Research and Treatment Center, King Saud UniversityRiyadh, Saudi Arabia; ^3^Psychological Sciences, University of MelbourneMelbourne, Australia; ^4^Departament de Psiquiatria i Psicobiologia Clínica, Facultat de Medicina, Universitat de BarcelonaBarcelona, Spain; ^5^Institut Universitari de Neurorehabilitació Guttmann, Universidad Autónoma de BarcelonaBadalona, Spain

**Keywords:** aging, motor system, cortico-spinal reactivity, cortico-spinal plasticity, navigated transcranial magnetic stimulation

## Abstract

Aging is associated with changes in the motor system that, over time, can lead to functional impairments and contribute negatively to the ability to recover after brain damage. Unfortunately, there are still many questions surrounding the physiological mechanisms underlying these impairments. We examined cortico-spinal excitability and plasticity in a young cohort (age range: 19–31) and an elderly cohort (age range: 47–73) of healthy right-handed individuals using navigated transcranial magnetic stimulation (nTMS). Subjects were evaluated with a combination of physiological [motor evoked potentials (MEPs), motor threshold (MT), intracortical inhibition (ICI), intracortical facilitation (ICF), and silent period (SP)] and behavioral [reaction time (RT), pinch force, 9 hole peg task (HPT)] measures at baseline and following one session of low-frequency (1 Hz) navigated repetitive TMS (rTMS) to the right (non-dominant) hemisphere. In the young cohort, the inhibitory effect of 1 Hz rTMS was significantly in the right hemisphere and a significant facilitatory effect was noted in the unstimulated hemisphere. Conversely, in the elderly cohort, we report only a trend toward a facilitatory effect in the unstimulated hemisphere, suggesting reduced cortical plasticity and interhemispheric communication. To this effect, we show that significant differences in hemispheric cortico-spinal excitability were present in the elderly cohort at baseline, with significantly reduced cortico-spinal excitability in the right hemisphere as compared to the left hemisphere. A correlation analysis revealed no significant relationship between cortical thickness of the selected region of interest (ROI) and MEPs in either young or old subjects prior to and following rTMS. When combined with our preliminary results, further research into this topic could lead to the development of neurophysiological markers pertinent to the diagnosis, prognosis, and treatment of neurological diseases characterized by monohemispheric damage and lateralized motor deficits.

## Introduction

Age-associated decline in muscle strength and motor performance has been well documented (Frontera et al., 1991; Clark and Manini, 2008). Recent functional and structural imaging studies have attempted to elucidate the physiological manifestations of these age-related changes (Goble et al., 2010; Seidler et al., 2010; Darbin, 2012; Heise et al., 2013; List et al., 2013). A consistent finding has been differential activation patterns across age groups during unimanual motor movements. Although all subjects tend to show activation of the contralateral motor cortex during motor tasks, younger-adults typically show a deactivation of the ipsilateral motor cortex whereas older-adults show a marked activation of the ipsilateral motor cortex during such tasks (Stefanovic et al., 2004; Riecker et al., 2006; McGregor et al., 2009).

Two major theories have emerged to explain this increase in ipsilateral motor cortical activation during unimanual motor movement with age. The first proposes that anatomical changes in the aging brain, most likely at the level of the corpus callosum, serve to block interhemispheric inhibitory signals sent by the heavily activated contralateral motor cortex. In support of this proposition are several studies which utilize both single- and paired-pulse transcranial magnetic stimulation (TMS) paradigms to explore interhemispheric communication (for review Fling et al., 2011). Findings have revealed that unimanual movements in younger adults do indeed lead to ipsilateral motor cortex inhibition (Kobayashi et al., 2003; Giovannelli et al., 2009) whereas similar actions do not generate a concomitant inhibition in older adults (Peinemann et al., 2001; Talelli et al., 2008).

The second theory proposes that exacerbated task burden in older-adults leads to a functional recruitment of bilateral motor cortices. This theory, originally proposed to account for differential activation during cognitive tasks, has recently been suggested as an explanation for the variation in motor activation across age ranges (Meister et al., 2005; Rajah and D'Esposito, 2005; Verstynen et al., 2005).

Repetitive transcranial magnetic stimulation (rTMS) is a tool well suited to aiding in the determination of whether older-adult ipsilateral activation is due to inhibitory signal disruption or active task recruitment. Suppression of motor evoked potentials (MEP) induced by suprathreshold magnetic stimulation has been demonstrated following slow frequency (1 Hz or less) rTMS applied for 5 min or more over the motor cortex (Chen et al., 1997; Maeda et al., 2000; Muellbacher et al., 2000). Known colloquially as “virtual lesions,” these prolonged neural disruptions can be used to determine the extent to which particular cortical regions contribute to a given task or activity.

Exploiting this virtual lesion paradigm, we examined cortico-spinal excitability and unimanual motor task performance in younger- and older-adults both before and after 26 min of inhibitory rTMS over the non-dominant motor cortex (ipsilateral to the dominantly recruited musculature). The non-dominant cortex was chosen because the majority of studies demonstrating a difference in ipsilateral motor cortical activation between young and old have made this determination using dominant motor activity (Stefanovic et al., 2004; Riecker et al., 2006; McGregor et al., 2009). If aged ipsilateral motor activation is caused by blocked inter-hemispheric inhibitory signals, we expected the rTMS would have little to no affect on motor performance in either population. However, if aged ipsilateral motor activation is due to functional recruitment, we expected the rTMS would disrupt and impair motor performance in the older population.

## Materials and methods

### Subjects

Research was approved by the human research ethics committee at Beth Israel Deaconess Medical Center. All work was reviewed and adhered to the standards put forward by the institutional review board.

Prior to undertaking the study, all participants provided informed consent. Eighteen healthy adults participated in this study. The delineation between young and old participants was set at 45 years of age. Ten were placed in the *young* cohort (range: 19–31 years; mean: 23.40 years) and eight were placed in the *old* cohort (range: 47–73 years; mean: 57.38 years, Table 1). All subjects were right handed as indexed by the Edinburgh Handedness Inventory (Oldfield, 1971) and exhibited normal cognitive status as indexed by the Mini Mental State Examination (Cockrell and Folstein, 1998). Neurological examinations revealed no signs suggestive of underlying neurological or psychological condition. Participants were not taking medications known to affect motor cortical excitability at the time of the study and none had contraindications to receive TMS (Rossi et al., 2009).

**Table 1:** **Demographic table**.

	**Young**	**Old**	***p*-value**
*n*	10	8	
Age (mean ± *SD*)	23.40 ± 3.50	57.38 ± 9.61	<0.001^*^
Gender (M/F)	6/4	3/5	0.637

### Experimental set-up

The stimulation setup consisted of a frameless stereotaxic system for navigation (Nexstim Ltd., Helsinki, Finland) equipped with a Nexstim 59 mm mean winding diameter figure-of-eight TMS coil and connected to a magnetic stimulator (MagPro, MagVenture A/S, Farum, Denmark) delivering biphasic pulses. Single and rTMS were both delivered using biphasic pulses delivered through the same coil. During stimulation, surface electromyography (EMG) was recorded and monitored continuously online (ME 6000, Mega Electronics Ltd., Kuopio, Finland) using pre-gelled, disposable Ag/AgCl electrodes (10 mm diameter). Active electrodes were attached to the skin overlying the first dorsal interosseus (FDI) muscle and reference electrodes were placed over the metacarpophalangeal joints. The EMG signals were filtered (8-500 Hz), amplified, displayed, and stored for off-line analysis. The TMS system delivered trigger pulses that synchronized the TMS and EMG systems.

During TMS measurements, the subjects sat in a comfortable recliner and held their hands supine on their laps. The subjects remained silent during the study to avoid speech-induced modulation of cortical excitability. The subjects were also monitored for drowsiness and asked to keep their eyes open throughout the experiment. Relaxation of the measured muscle was controlled by continuous visual and audio EMG monitoring.

### Mapping protocol

**Figure 1:**
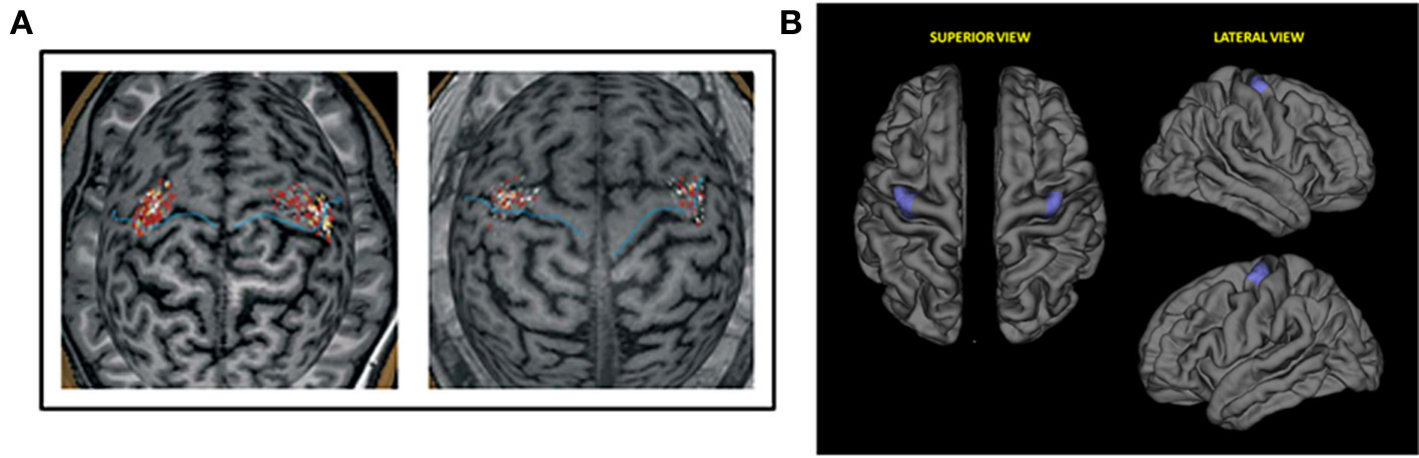
**(A)** Representative first dorsal interosseus (FDI) motor evoked potentials (MEPs) data for study sample. Single pulses applied to the FDI hotspot on MR images of the subjects' brains. Left: young subject (aged 23 years); Right: older subject (aged 60 years). **(B)** Region of interest (ROI) selected for the correlation analysis of cortical thickness and motor evoked potentials. M: Medial; A: Anterior; P: Posterior; L: Lateral.

Prior to TMS, all subjects underwent a high-resolution T1-weighted structural MRI scan. Imaging data were fed to the navigation software (eXimia 3.1, Nexstim Ltd., Helsinki Finland) for automatic 3D brain reconstruction that was used to guide navigation and deliver TMS over M1 (“hot spot”). In each session, the motor cortical output was mapped carefully for the optimal representation of the FDI muscle on both hemispheres (Figure 1A).

We determined the optimal scalp positions and lowest stimulation intensity capable of eliciting MEPs in the contralateral FDI muscle. According to the recommendations of the International Federation for Clinical Neurophysiology, motor threshold (MT) was defined as the lowest stimulator output intensity that produced at least five MEPs out of 10 consecutive stimuli of at least 50 μ V peak-to-peak amplitude (Rossi et al., 2009). The absence of background activity was monitored on-line with continuous visual monitoring of spontaneous EMG.

### TMS

#### Absolute silent period

Absolute cortical silent period (SP) (short duration of EMG suppression following MEP elicitation) was obtained under resting conditions during a submaximal isometric finger and thumb contraction at intensity of approximately 20% of maximal voluntary contraction (MVC) strength. Specific details on these procedures are provided in previous study (Bashir et al., 2010).

#### Paired-pulse paradigms

Paired-pulse paradigms included a subthreshold conditioning stimulus (CS: 90% of rMT), and a suprathreshold test stimulus (TS: 120% of rMT) with two different inter stimulus intervals (ISI: 3 and 12 ms). Ten TS evoked responses were collected for each CS intensity and each ISI condition. Non-conditioned (control) MEP response was determined from ten stimuli at the TS intensity (120% of MT) (Bashir et al., 2010).

#### Single-pulse TMS and rTMS intervention

Ten single stimuli (4–8 s apart) were delivered to the right FDI hot spot at an intensity of 120% of rMT to determine baseline MEP amplitude and latency.

All subjects then received a train of 1600 stimuli of 1 Hz rTMS at 90% rMT to the right (non-dominant) M1. rTMS was delivered over the optimal site for induction of MEPs in the left FDI muscle. rTMS in the 1 Hz range has previously been shown (in most subjects) to suppress excitability of the motor cortex in the targeted hemisphere and increase excitability in the contralateral motor cortex (reviewed by Kobayashi and Pascual-Leone, 2003; Fregni and Pascual-Leone, 2007). During the delivery of rTMS, the 3D brain of the participant's cortex and the hot spot were visualized with the navigated brain stimulation software for optimal delivery.

**Figure 2:**
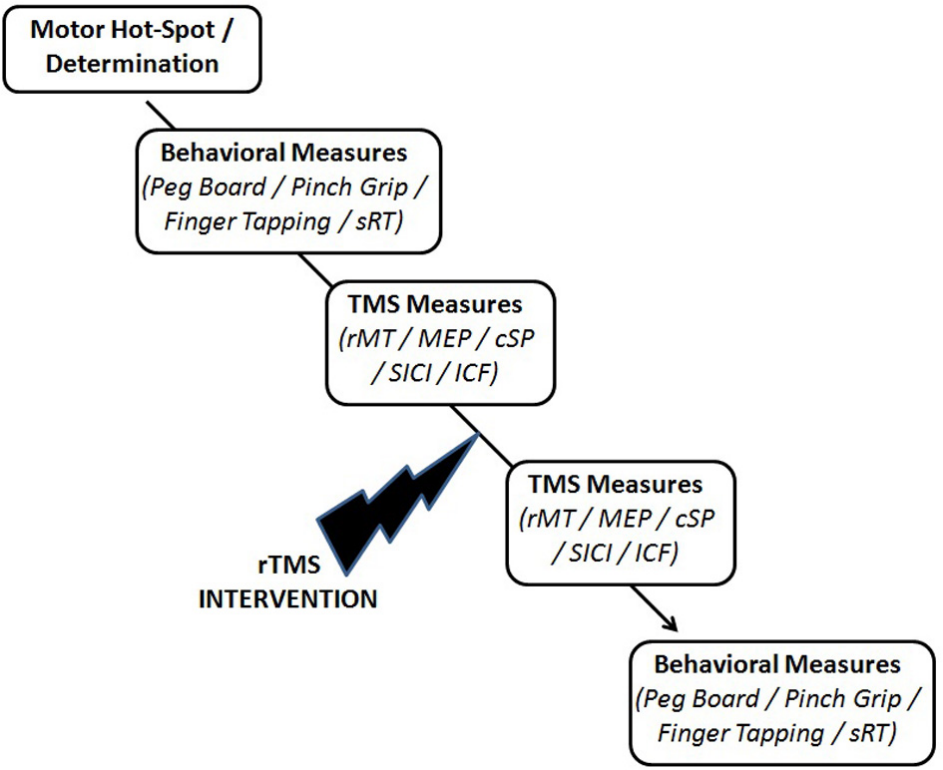
**Experimental testing schematic**.

Immediately following the rTMS intervention, the prior TMS measures were re-evaluated (Figure 2).

### Behavioral tasks

Prior to and following the rTMS intervention, participants undertook a battery evaluation of both right and left hand motor function. Behavioral tasks were administered immediately following the neurophysiological measurement of rMT. Tasks were presented in the following order with the right hand tested before the left hand.

#### Nine-hole peg test

The time required for subjects to insert nine pegs into a pegboard and then remove the pegs from the filled pegboard was recorded across three trials. A 1-min break was given between each trial.

#### Pinch grip strength

Average pinch grip strength (kg force) was measured according to a previously described protocol that exhibits good validity and test-retest reliability (Mathiowetz et al., 1984). Subjects grasped a pinch key with the pad of the thumb opposed against the lateral aspect of the middle phalanx of the index finger. Subjects performed three pinch grip trials, lasting 10 s each, with 1 min of rest between trials.

#### Finger tapping task

Subjects were asked to tap a button with their index finger of each hand as quickly as possible for the duration of 10 s. The tapping assessment comprised five trials with 15-s of rest between each trial (with the exception of a 1-min break between the third and fourth trials).

#### Simple reaction time task

Subjects were instructed to press the space bar on a common keyboard with their index finger as fast as possible in response to an on-screen stimulus. The time between stimulus onset and response was recorded. Two trials of 20 stimuli each were presented to each participant prior to and after rTMS.

### MRI analysis of cortical thickness

Cortical reconstruction and analysis of participants' structural MRIs was run with the FreeSurfer image analysis suite (http://surfer.nmr.mgh.harvard.edu), which is documented and freely available for download online (http://surfer.nmr.mgh.harvard.edu/). The technical details of these procedures are described in prior publications (Fischl and Dale, 2000). Briefly, this processing includes motion correction and averaging of multiple volumetric T1 weighted images, removal of non-brain tissue, intensity normalization (Sled et al., 1998), tessellation of the gray matter white matter boundary, automated topology correction (Ségonne et al., 2007), and surface deformation following intensity gradients to optimally place the gray/white and gray/cerebrospinal fluid borders at the location where the greatest shift in intensity defines the transition to the other tissue class (Fischl and Dale, 2000). Once the cortical models were complete, reconstructed and registered individual cortical thickness maps were smoothed using a Gaussian kernel of 10 mm full-width half maximum and subsequently used in statistical analyses. In order to assess if cortical thickness could explain the differences in plasticity measured by MEPs we performed a correlation analysis between subjects' MEPs and the values of cortical thickness of a region of interest (ROI) (Figure 1B). This particular ROI was drawn to encompass the hot spots of all subjects. The ROI was done firstly in standard space-brain and then was mapped for each subject in order to measure this region-specific for each individual.

### Data and statistical analyses

For MEP determination in response to TMS, continuous EMG was sampled to 350 ms epochs, 50 ms before and 300 ms after each TMS pulse. The latencies were marked manually by visual inspection and determination of the onset of the MEP. FDI muscle responses were analyzed using MegaWin software (Mega Electronics Ltd., Kuopio, Finland). Cortico-spinal excitability was assessed by measuring peak-to-peak amplitude of MEPs in the contralateral FDI muscle in response to single TMS pulses. To minimize the variability of TMS-induced single-pulse responses, the largest and smallest MEP amplitude responses were excluded from analysis—no additional single-pulse MEP responses were excluded. Behavioral measures, for the respective hand both before and after rTMS, were averaged across all trials performed by each participant. Additionally, we computed the percent change (%Δ) in MEP amplitude pre- to post-rTMS. As young and elderly populations are independent and a normal distribution cannot be assumed, interhemispheric and pre/post-TMS measures were compared using the nonparametric Wilcoxon signed rank test.

Cortico-spinal excitability was assessed by measuring peak-to-peak amplitude of MEPs in the contralateral FDI muscle in response to single TMS pulses. MEP amplitude and SP duration were determined as the mean response of 10 trials in each subject. The percent change in MEP (MEP %Δ) as a result of paired-pulse relative to the non-conditioned response were computed and used in the subsequent analyses. We also computed a MEP defined as (MEP %Δ after rTMS/MEP %Δ before rTMS) for each ISI and each hemisphere, which we used in correlation analysis.

To compare the distribution of the ages of subjects between the young and elderly groups, we used a two-sample Kolmogorov-Smirnov goodness-of-fit hypothesis test. To compare groups on the binary classification of gender, we used Fisher's exact test. A Mann-Whitney U-test was used to compare between the younger and the older groups for all additional measures. All statistical tests were two-tailed, with statistical significance defined at *p* < 0.05. All correlations were performed using Spearman's rank correlation. All statistical analyses were performed using MatLab (version 7.4.0).

## Results

### Between-group analyses of demographic data and cortical thickness

There were no differences in gender, EHI handedness scores, or MMSE scores between the groups (Table 1).

**Figure 3:**
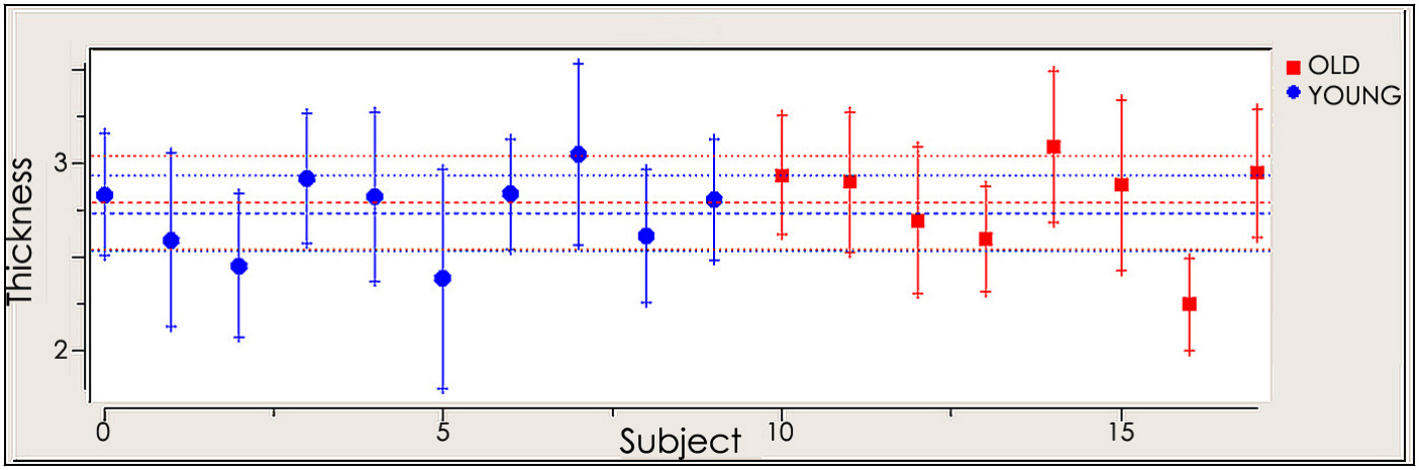
**Cortical thickness of selected region of interest (ROI) in young and old cohort**.

To assess cortical thickness as a potential confounding factor, we performed a correlation analysis using the cortical thickness maps of the targeted right M1, reconstructed from the subjects' anatomical MRIs. Analysis of whole brain cortical thickness maps revealed no significant differences of cortical thickness between young and old subjects. The correlation analysis revealed no significant relationship between the cortical thickness of the selected ROI and MEPs before and after rTMS in the young (before rTMS: *r* = −0.154, *p* = 0.672; after rTMS: *r* = −0.219, *p* = 0.544) and old (before rTMS: *r* = 0.455, *p* = 0.257; after rTMS: *r* = 0.221, *p* = 0.600), thus suggesting that there are no differences in cortical thickness confounding the results presented (Figure 3).

### Baseline neurophysiologic characteristics

**Figure 4:**
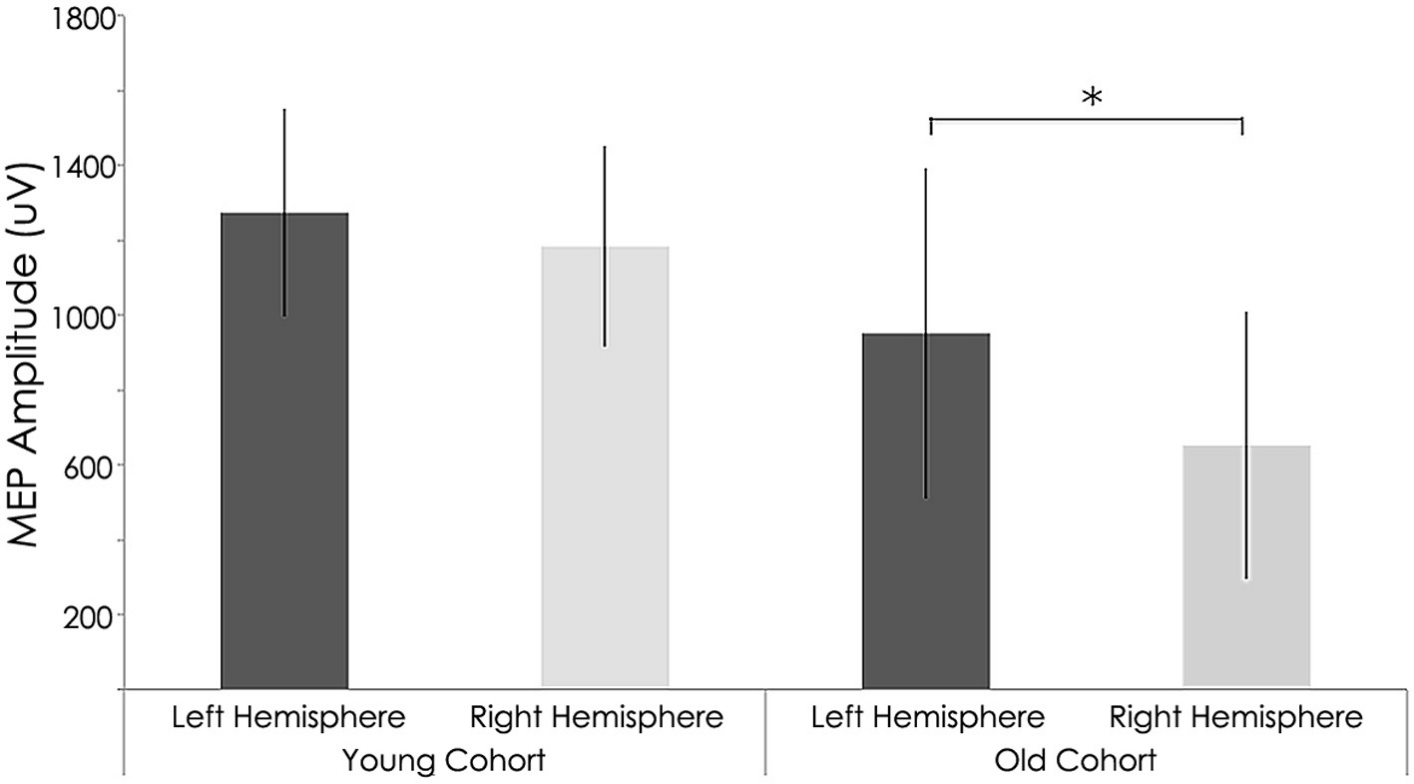
**Interhemispheric characteristics of the study sample prior to rTMS**. Non-significant statistical differences in Motor evoked potentials (MEPs) between right and left hemispheres among young subjects (*p* = 0.122; Wilcoxon signed-rank test); Statistically significant differences between right and left hemispheres among older subjects (*p* = 0.004; Wilcoxon signed-rank test). ^*^, statistically significant (*p* < 0.05).

At baseline, older subjects exhibited significantly higher resting motor thresholds (RMT) than younger subjects in the non-dominant (right) hemisphere (young adults: 40.15 ± 4.48%; old adults: 46.71 ± 6.29% of maximum stimulator output; *p* = 0.014). Moreover, the amplitude of MEP elicited from the right hemisphere was significantly reduced in older subjects (young group: 1184.24 ± 267.76 mV; old group: 652.35 ± 359.08 mV; *p* = 0.004, Table 2 and Figure 4). For the left (dominant) hemisphere, there was a trend toward increased RMT and smaller MEP amplitude in the older group as compared with the young cohort, but it did not reach significance (RMT—young group: 40.00 ± 5.96%; old group: 46.26 ± 6.21%; *p* = 0.058—MEP—young group: 1274.72 ± 278.92 mV; old group: 952.37 ± 442.10 mV; *p* = 0.122, Table 2 and Figure 4).

**Table 2:** **Measures of bilateral cortio-spinal reactivity and plasticity**.

	**Motor threshold [% Stimulator output]**		**MEP amplitude [μV]**	
	**Young**	**Old**	**Young**	**Old**
**BEFORE rTMS**				
Left hemisphere	40 ± 5.95	46 ± 6.2	1274 ± 278	952 ± 442
Right hemisphere	40 ± 4.79	46.7 ± 6.29	1184 ± 267	652 ± 359
**AFTER rTMS**				
Left hemisphere	41 ± 6.0	44 ± 5.37	1670 ± 403	1227 ± 811
Right hemisphere	40 ± 6.0	49 ± 9.38	1040 ± 266	647 ± 496

*Data are presented as mean ± standard deviation*.

### Intracortical excitability

**Figure 5:**
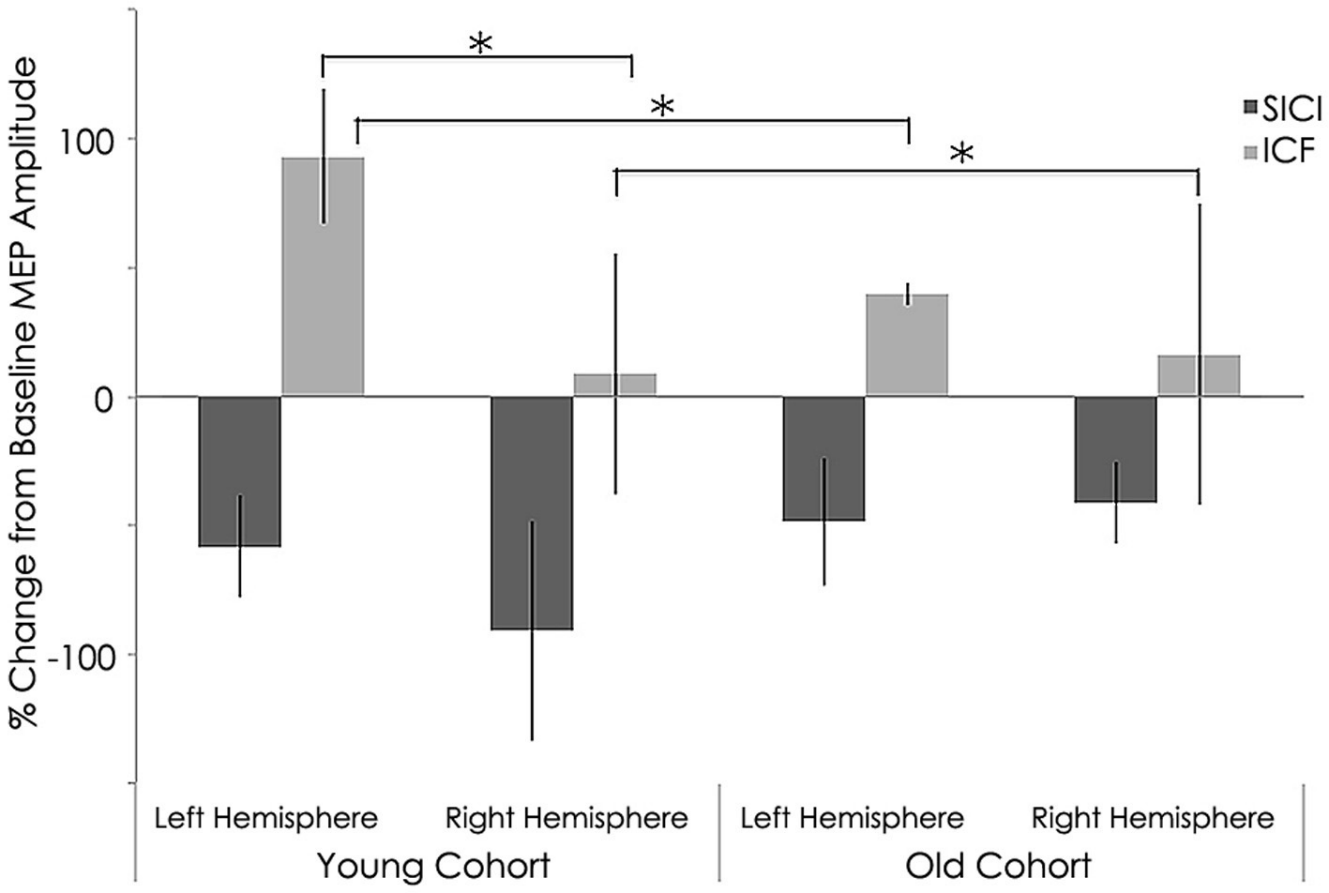
**Interhemispheric characteristics of the study sample prior to rTMS for short intracortical inhibition (SICI) and intracortical facilitation (ICF)**. (SICI) at interstimulus intervals of 3 ms and ICF at 12 ms (normalized and express as 100% level). Significant statistical differences in MEPs between left (*p* = 0.030) and right (*p* = 0.01) hemispheres among young subjects for ICF condition significant difference between hemispheres (*p* = 0.021) for young subjects; ^*^, statistically significant (*p* < 0.05).

Intracortical excitability measured as short intracortical inhibition (SICI) and intracortical facilitation (ICF). MEP %Δ pre-rTMS for 3 ms ISI (SICI) was not significant between the young and old cohorts in the left (young: −58.28 ± 19.69 mV; old: −42.27 ± 29.12 mV; *p* = 0.183) or right hemisphere (young: −40.82 ± 42.75 mV; old: −27.39 ± 41.90 mV; *p* = 0.514, Figure 5). However, MEP %Δ pre-rTMS for 12 ms ISI (ICF) was significant between the young and old cohorts in the left (young: −15.91 ± 53.89 mV; old: 43.34 ± 50.86 mV; *p* = 0.030) and right hemispheres (young: 129.10 ± 95.31 mV; old: 18.05 ± 54.35 mV; *p* = 0.010, Figure 5).

### Motor performance behavioral data

**Figure 6:**
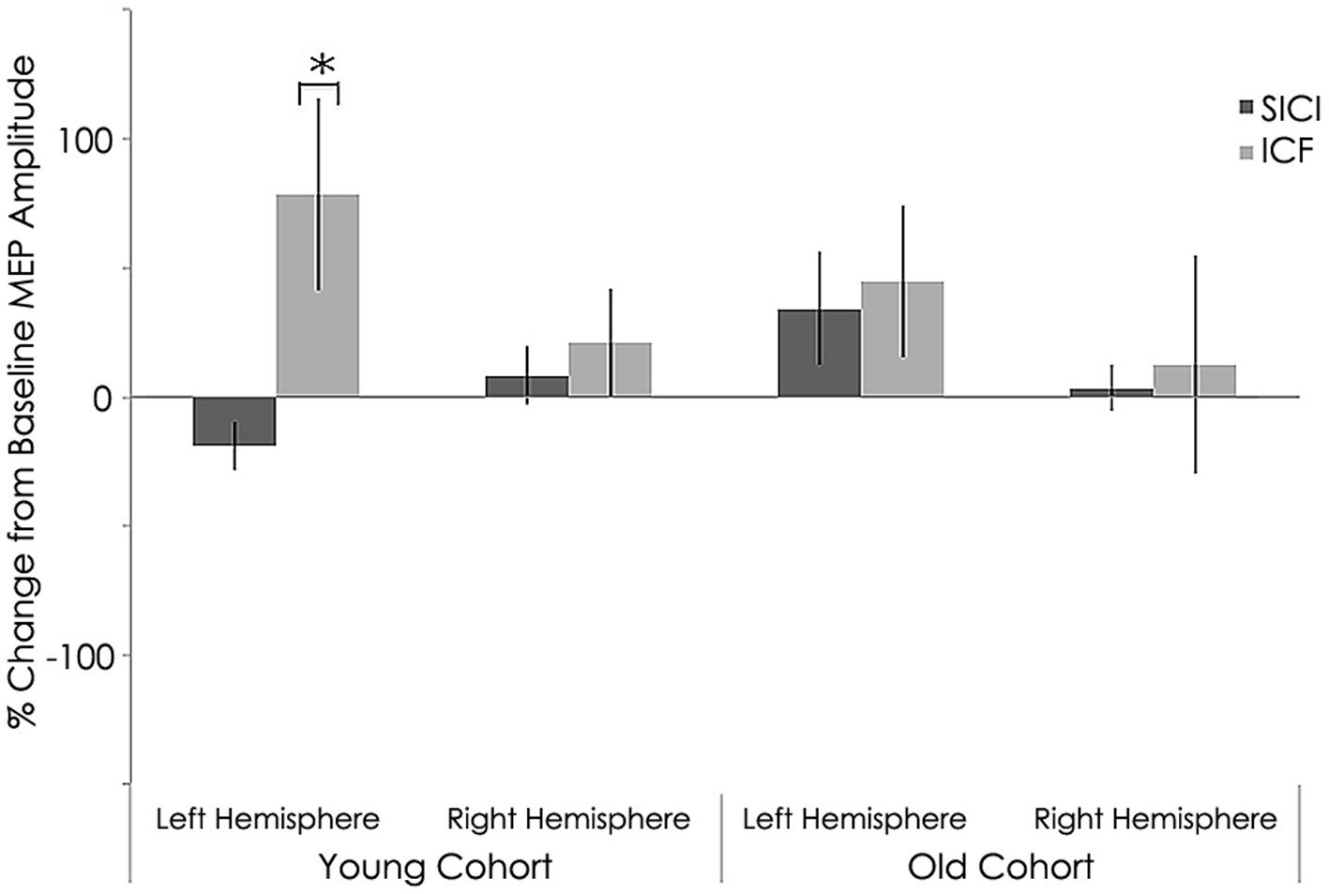
**Interhemispheric characteristics for short intracortical inhibition (SICI) and intracortical facilitation (ICF)**. (SICI) at interstimulus intervals of 3 ms and ICF at 12 ms (normalized and express as 100% level) of the study sample after rTMS. Statistically significant increased in in MEPs for intracortical facilitation (ICF) for left hemispheres among young subjects (*p* = 0.002; Wilcoxon signed-rank test); ^*^, statistically significant (*p* < 0.05).

At baseline, we found a significant group difference in the reaction time (RT) and finger tapping tasks only (Tables 3, 4). RTs for the older group were significantly longer than for the younger group in both the left (young group: 486.43 ± 45.19 ms; old group: 608.88 ± 63.70 ms; *p* = 0.001, Tables 3, 4 and Figure 6) and right hands (young group: 510.56 ± 57.69 ms; old group: 615.67 ± 97.02 ms; *p* = 0.012, Figure 6). The number of taps recorded for the older group was significantly reduced compared to that of the younger subjects in both the left (young group: 44.60 ± 4.27; old group: 33.65 ± 4.80; *p* = 0.001) and right hands (young adults: 46.10 ± 5.09; old adults: 35.73 ± 5.81; *p* < 0.001). With regards to the 9-hole peg task, there was only a significant difference at baseline (*p* = 0.043) between the two groups (Tables 3, 4).

**Table 3:** **Pre- and Post-rRMS comparisons of behavioral data for younger subjects**.

	**Before rTMS**	**After rTMS**	***p*-value**
**REACTION TIME**			
Left hand	486.43 ± 45.19	542.42 ± 43.22	0.002^*^^iv^
Right hand	510.56 ± 57.69	486.35 ± 41.48	0.193
**PINCH GRIP**			
Left hand	27.00 ± 10.56	29.30 ± 11.54	0.072
Right hand	28.30 ± 10.87	31.50 ± 9.70	0.074
**FINGER TAPPING**			
Left hand	44.60 ± 4.27	44.90 ± 4.43	0.941
Right hand	46.10 ± 5.09	47.45 ± 4.81	0.106
**9-HOLE PEG**			
Left hand	18.98 ± 2.16	19.20 ± 2.23	0.281
Right hand	18.42 ± 2.28	18.60 ± 2.12	0.641

*Data are presented as mean ± standard deviation*.

iv , Wilcoxon signed-rank test;

* *, statistically significant (p < 0.05)*.

**Table 4:** **Pre- and Post-rRMS comparisons of behavioral data for older subjects**.

	**Before rTMS**	**After rTMS**	***p*-value**
**REACTION TIME**			
Left hand	608.88 ± 63.70	640. 34 ± 70.36	0.016^*^^iv^
Right Hand	615.67 ± 97.02	601.80 ± 72.86	0.414
**PINCH GRIP**			
Left hand	26.42 ± 10.55	27.58 ± 11.76	0.250
Right hand	27.13 ± 11.81	31.21 ± 11.73	0.008^*^^iv^
**FINGER TAPPING**			
Left hand	33.65 ± 4.80	31.48 ± 4.81	0.141
Right hand	35.73 ± 5.81	35.23 ± 5.86	0.219
**9-HOLE PEG**			
Left hand	22.23 ± 3.87	22.55 ± 3.60	0.297
Right hand	22.54 ± 4.57	22.31 ± 4.56	0.867

*Data are presented as mean ± standard deviation*.

iv , Wilcoxon signed-rank test;

* *, statistically significant (p < 0.05)*.

### Effect of rTMS on neurophysiologic characteristics

**Figure 7:**
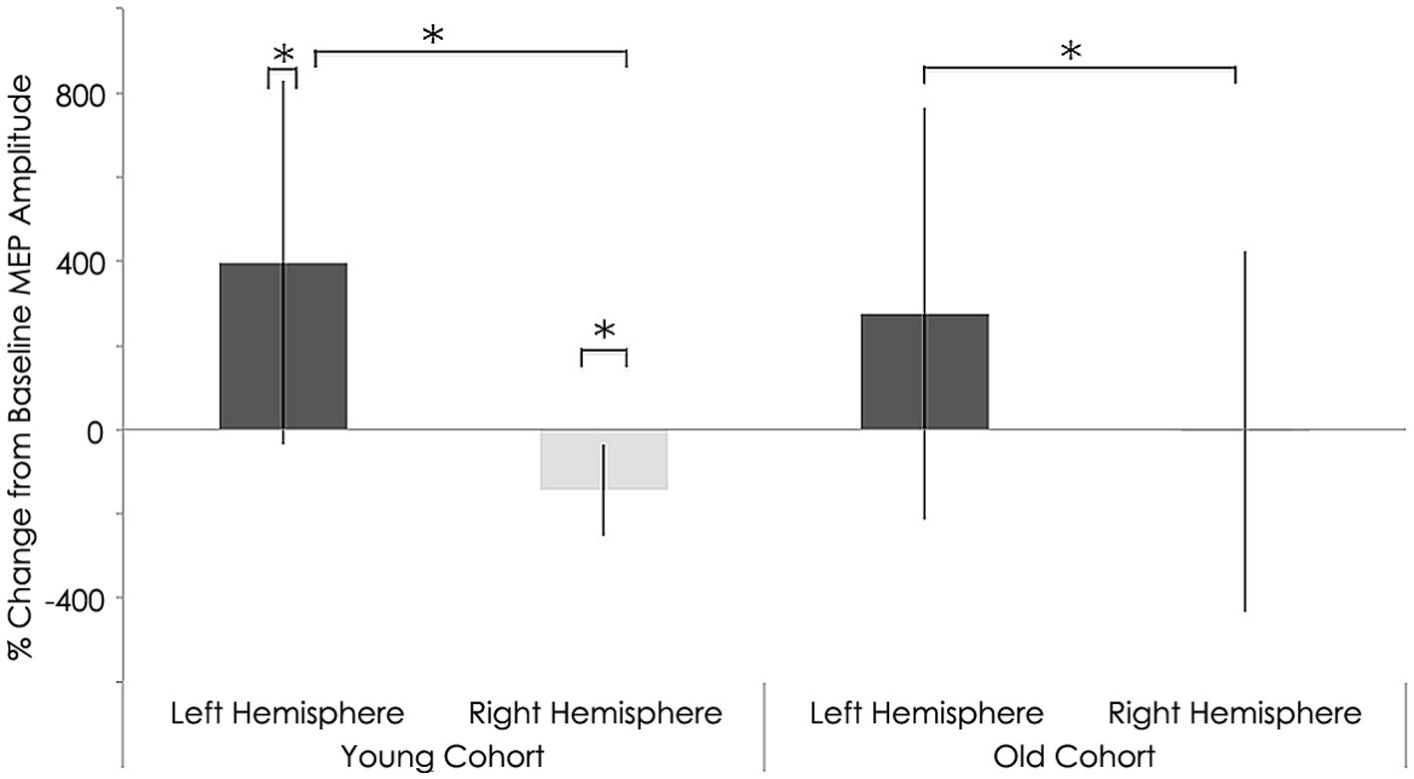
**Interhemispheric characteristics of the study sample after rTMS**. Statistically significant differences in motor evoked potentials (MEPs) between right and left hemispheres among young subjects (*p* = 0.037; Wilcoxon signed-rank test); Significant differences between right and left hemispheres among older subjects (*p* = 0.04; Wilcoxon signed-rank test). Significant increase in MEPs in left (*p* = 0.01) and decrease in right (*p* = 0.010) hemispheres in young subjects; ^*^, statistically significant (*p* < 0.05).

After rTMS to the right hemisphere, only the younger adults exhibited significant interhemispheric differences in evoked MEP amplitudes (Figure 7). That is, significant differences between the left and right hemispheres for the younger group were detected after 1 Hz navigated rTMS, although such a significant interhemispheric difference for the younger group was not present at baseline (Figure 4). After rTMS, MEPs elicited from the left (unstimulated) M1 increased significantly for the young adults (*p* = 0.037). Conversely, MEPs from the right (stimulated) M1 decreased significantly (*p* = 0.010) in the young subjects. The effect of rTMS was not significant in either the left or right hemisphere in the old group (*p* = 0.414 and *p* = 0.982, respectively); although the pattern of left M1 MEP amplitude increase/right M1 MEP amplitude decrease pattern was present (Table 2).

With regards to RMT and MEP shifts from baseline values, a couple significant group differences were found. Relative to the young, the older adults exhibited significantly elevated RMT (young group: 40.50 ± 6.06%; old group: 49.38 ± 9.38% of maximum stimulator output; *p* = 0.028, Table 2) and significantly lower MEP amplitudes (young group: 1040.62 ± 266.34 mV; old group: 647.75 ± 496.48 mV; *p* = 0.011, Figure 7) in the contralateral (non-dominant) hand. In the ipsilateral (dominant) hand, change in RMT and MEP were not significantly different between the young and old groups (RMT—young group: 41.00 ± 5.31%; old group: 44.63 ± 5.37% of maximum stimulator output; *p* = 0.300: MEP—young group: 1670.84 ± 403.21 mV; old group: 1227.50 ± 811.15 mV; *p* = 0.068, Figure 7).

#### Effect of rTMS on intracortical excitability

Similar to baseline, MEP %Δ post-rTMS for 3 ms ISI (SICI) was not significant between the young and old cohorts in the left (young: −36.94 ± 38.33 mV; old: −25.21 ± 28.40 mV; *p* = 0.482) or right hemisphere(young: −54.27 ± 8.91 mV; old: −0.66 ± 88.25 mV; *p* = 0.072, Figure 6). Interestingly, unlike at baseline, MEP %Δ post-rTMS for 12 ms ISI (ICF) was not significant between the young and old cohorts in the left (young: 32.54 ± 75.01 mV; old: 118.94 ± 171.74 mV; *p* = 0.170) or right hemisphere (young: 66.14 ± 54.13 mV; old: 153.85 ± 170.67 mV; *p* = 0.143, Figure 6). Although these differences did not reach significant, they are rather large. It is possible our relatively small sample size contributed to this statistical outcome. Additional participants could help clarify the significance of these differences.

### Effect of rTMS on absolute silent period

**Figure 8:**
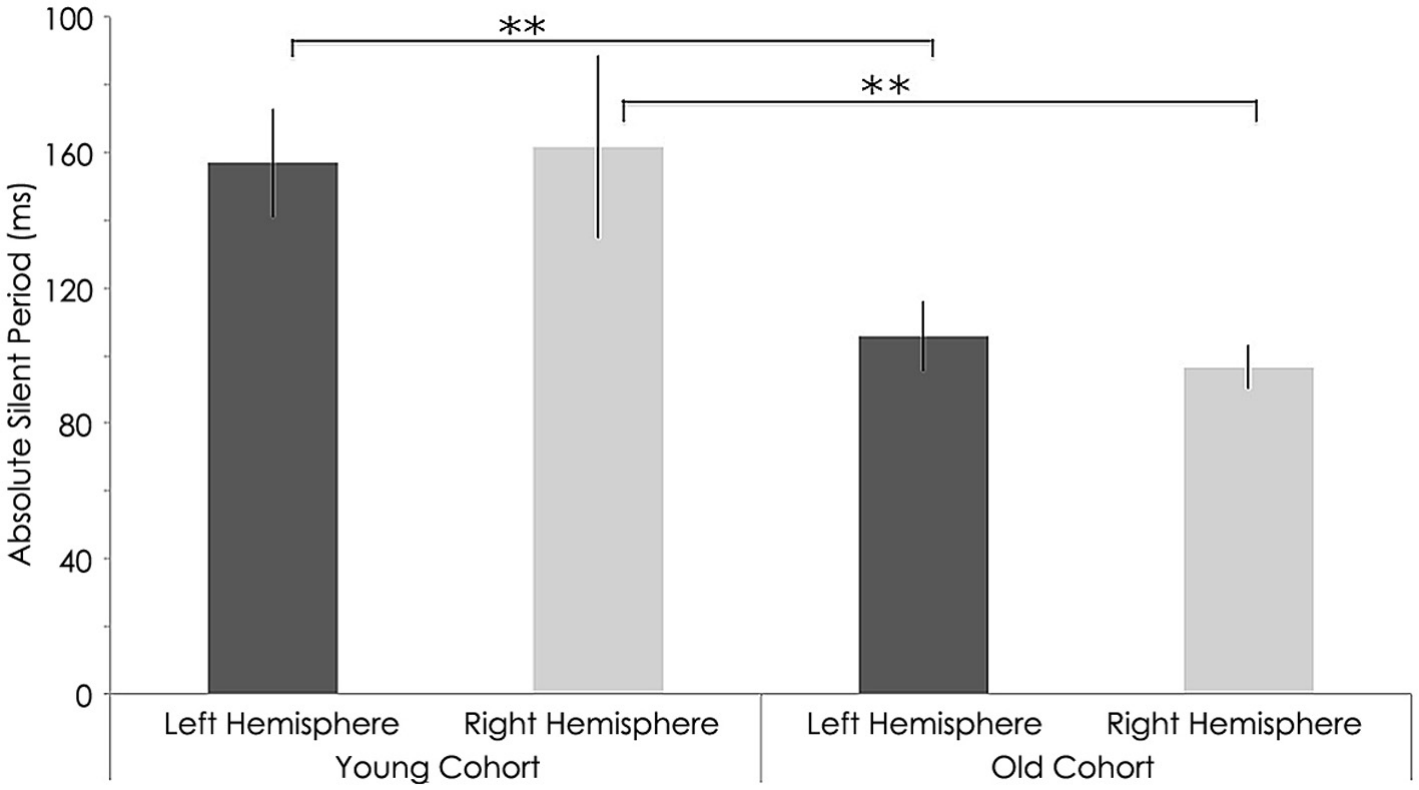
**Interhemispheric characteristics for absolute SP measure in (ms) of the study sample prior to rTMS**. significant statistical differences in absolute silent period in left hemispheres among young and old subjects (*p* = 0.001; Wilcoxon signed-rank test); Statistically significant differences in right hemispheres between young and older subjects (*p* = 0.001; Wilcoxon signed-rank test).^**^, statistically significant (*p* < 0.05).

**Figure 9:**
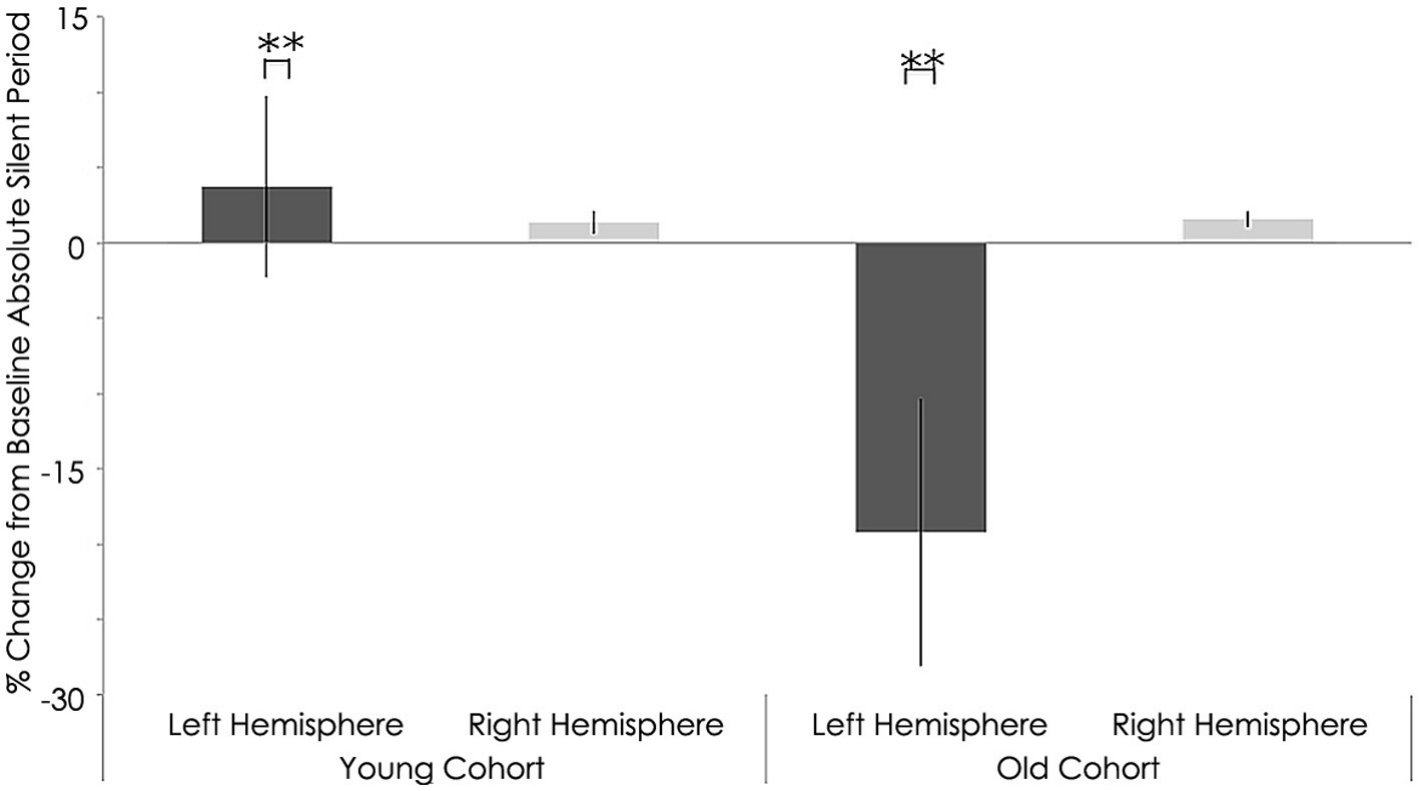
**Interhemispheric characteristics measure as absolute silent period (ms) of the study sample after rTMS**. Statistically significant increased in absolute silent period among young subjects (*p* = 0.001; Wilcoxon signed-rank test); Significant decreased in left hemispheres among older subjects (*p* = 0.001; Wilcoxon signed-rank test).^**^, statistically significant (*p* < 0.05).

Relative to the young cohort, the old cohort exhibited significantly shorter absolute SP duration in the left hemisphere, both before (young: 157.05 ± 16.10 ms; old: 105.76 ± 10.29 ms; *p* < 0.001, Figure 8) and after rTMS (young: 160.81 ± 22.13 ms; old: 86.54 ± 11.99 ms; *p* < 0.001, Figure 9). Significantly shorter absolute SP duration was also noted for the old cohort in the right hemisphere, both before (young: 161.66 ± 26.92 ms; old: 96.48 ± 6.41 ms; *p* < 0.001, Figure 7) and after (young: 163.03 ± 26.16 ms; old: 98.08 ± 5.84 ms; *p* < 0.001) rTMS (Figure 9).

### Functional significance of rTMS as assessed by motor performance

**Figure 10:**
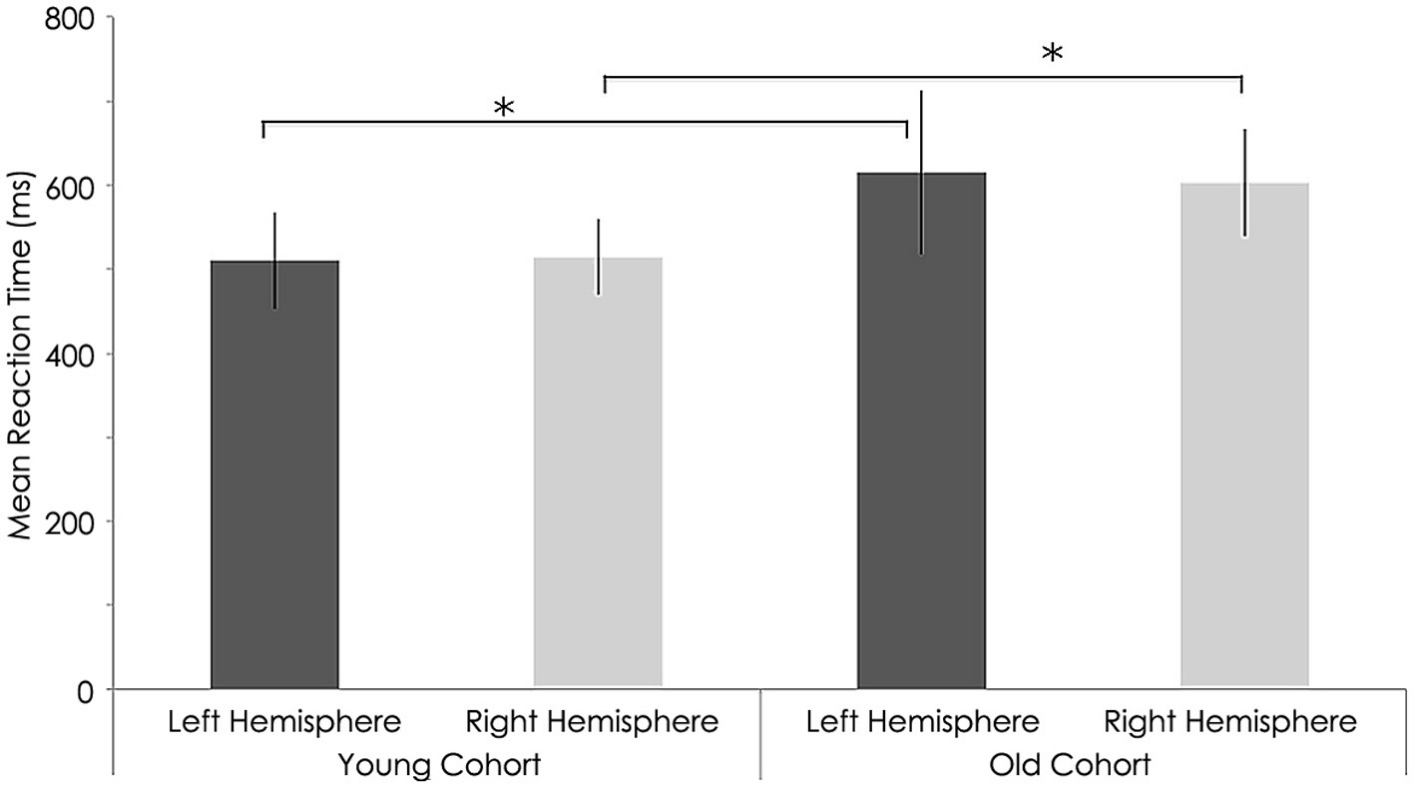
**Behavioral characteristics of the study sample before rTMS for reaction time (ms) task**. Statistically significant differences in reaction time between right hemispheres (*p* = 0.012) and left hemisphere (*p* = 0.01, Wilcoxon signed-rank test) among young and older subjects. ^*^, statistically significant (*p* < 0.05).

**Figure 11:**
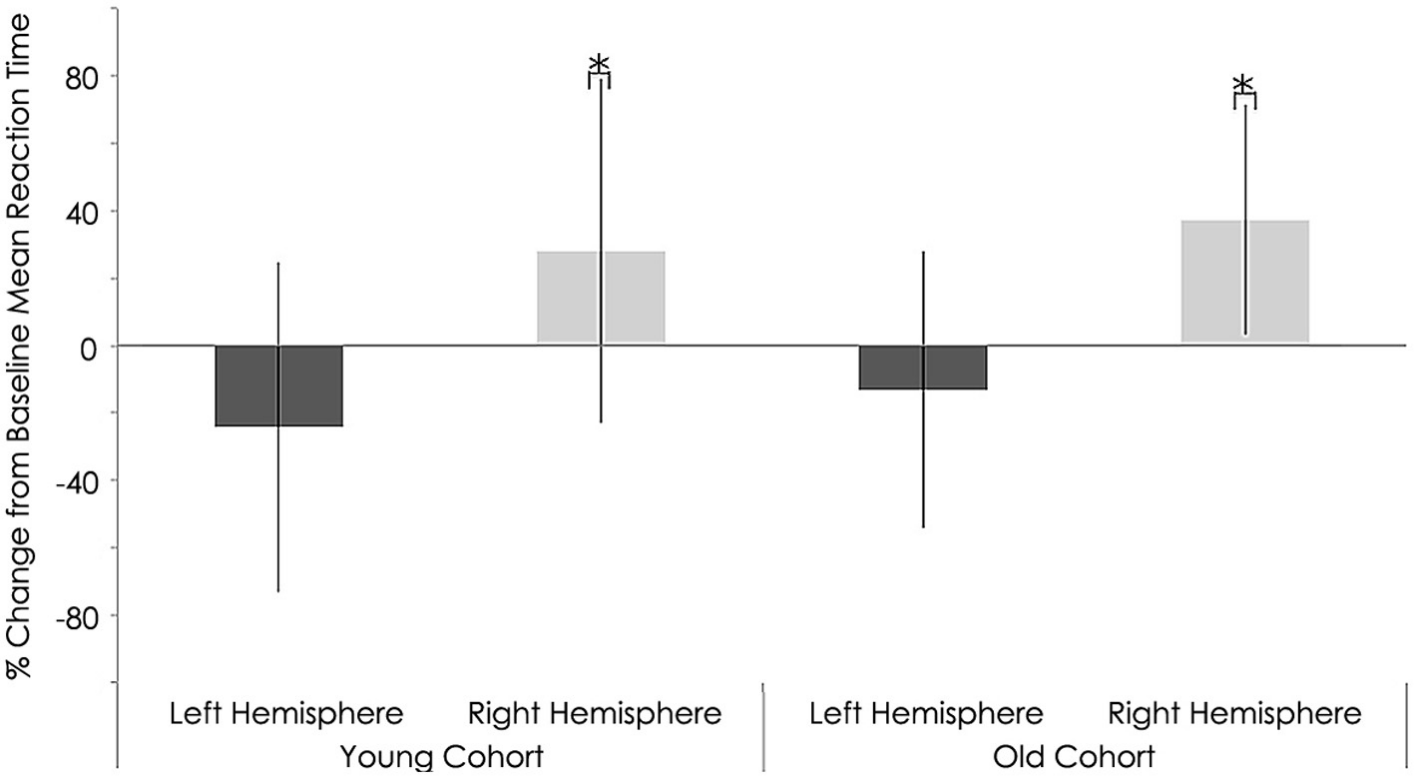
**Behavioral characteristics of the study sample after rTMS for reaction time (ms) task**. Statistically significant increase in reaction time right hemispheres among young (*p* = 0.002) and older subjects (*p* = 0.016; Wilcoxon signed-rank test).^*^, statistically significant (*p* < 0.05).

Neither the young nor old cohorts showed any significant changes from baseline with regards to RT with the right (non-stimulated hemisphere) hand (Young: *p* = 0.144, Old: *p* = 0.193). As at baseline, this measure showed a significant difference between groups (*p* = 0.003, Tables 3, 4). With the left (stimulated hemisphere) hand, both groups showed increased RT following rTMS as compared to baseline (Young: *p* = 0.002, Old: *p* = 0.016, Tables 3, 4, Figures 10, 11). The left hand also showed a significant difference between groups both at baseline (*p* = 0.000)- and after rTMS (*p* = 0.006).

For the grip strength task, there was a significant increases in the right hand of the elderly cohort following stimulation (*p* = 0.008). No effects were found for either hand in either cohort for the finger tapping or 9-Hole Peg tasks.

### Pilot data about the effects of age on motor cortical excitability

**Figure 12:**
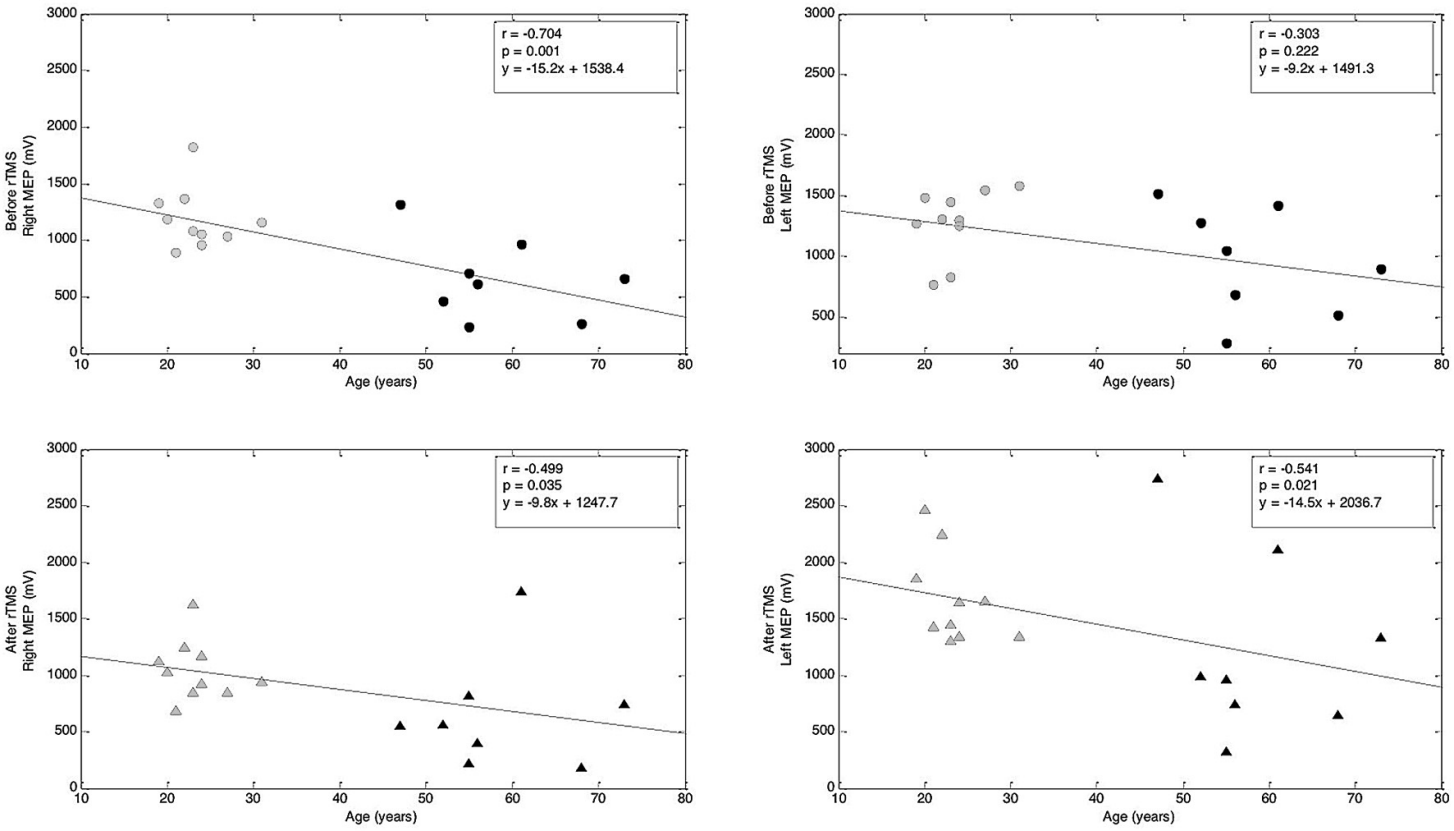
**Correlations between age and MEP amplitude before and after rTMS**. Top row: Correlation between age and MEP amplitude before rTMS to M1. Bottom row: Correlation between age and MEP amplitude after rTMS to M1. Correlation coefficients (r) and corresponding *p*-values are indicated on the figure (Spearman's rank correlation). Regression lines are shown; corresponding regression slope and intercept values are indicated on the figures.

We scrutinized the motor cortical excitability of each hemisphere across the lifespan by computing correlations between MEP amplitude and age. At baseline, we found a strongly negative, significant correlation between the MEP amplitudes and age in the right hemisphere (*r* = −0.704, *p* = 0.001; Spearman's rank correlation). However, in the left hemisphere, we found a weakly negative, non-significant association (*r* = −0.303, *p* = 0.222; Spearman's rank correlation, Figure 12). These correlations suggest that, as one ages, baseline MEP amplitude shows a significant decrease in the right hemisphere with a less-prominent and non-significant decrease in the left hemisphere.

Lastly, when analyzing the correlation between MEP amplitude and age after the rTMS intervention, we found negative and significant correlations in both the left (*r* = −0.541, *p* = 0.021; Spearman's rank correlation) and right (*r* = −0.499, *p* = 0.035; Spearman's rank correlation) hemispheres (Figure 12). These correlations suggest that, following 1 Hz rTMS, MEP amplitude in both hemispheres decreases across the lifespan.

## Discussion

### Overview of findings

In this study, we investigated the effect of age on the response to navigated 1 Hz rTMS applied over non-dominant M1. A main finding was that the effect of rTMS on MEP amplitudes evoked in the right FDI by single pulse TMS to both the left and right motor cortices significantly increased and decreased, respectively, as compared to the MEP amplitudes recorded at baseline. For the old group, pre- and post-rTMS MEP measures did not differ significantly. The most novel findings of this work are that older adults exhibited less ICF and more intracortical inhibition (ICI). We also observed that older adults had a reduced SP compared to young cohort group.

Additionally, we found reduced motor cortex excitability, reduced cortical inhibition, and minimal task-related modulation of corticospinal responses in the non-dominant hand of older-adults as compared to younger subjects. In contrast, we found increased task-dependent modulation of the corticospinal pathway in the dominant hand of older adults. These differences in the corticospinal control in the right and left hands of older adults may be reflective of neural changes brought about by many years of one-hand preference during skilled motor tasks.

We also observed increased SICI and a longer SP in older adults. This indicates increased GABAergic inhibition with age under resting conditions. Additionally, we found a decreased ICF in older adults. This could be the result of the inhibitory increase or a wholly separate facilitatory (glutamatergic) decrease.

### Motor performance changes following rTMS

Behaviorally, there were significant increases for both the young and old groups in the RT task in the left (contralateral) hand following rTMS. This result is consistent with the neurophysiologic findings of a greater inter-hemispheric impact of 1 Hz rTMS (Avanzino et al., 2008). Interestingly, although both cohorts showed faster RTs in the ipsilateral, non-stimulated hand, neither reached statistical significance.

### Reduced corticospinal excitability in aged subjects

A major finding concerned the effect of rTMS on MEP amplitudes evoked by single-pulse TMS to both the left and right motor cortices. As compared to pre-rTMS baseline measurements, the younger population showed a significant increase in dominant hand MEP values and a significant decrease in non-dominant hand MEP values. Interestingly, the older population showed no significant MEP differences in either hand.

These findings confirm recent results reported by Todd et al. (2010) who report that a group of younger subjects exhibited significantly reduced MEP amplitude in response to 10 min of real 6 Hz rTMS as compared to sham stimulation, but that a group of older subjects showed no such differences. These results are also consistent with results recently reported by Rogasch et al. (2009) who showed that MEP amplitudes in response to a thumb training task significantly increased in younger adults, but not in older adults. Together, these findings suggest that the reduced effect of low-frequency rTMS on MEP size in older adults reflects a decrease in both basal and use-dependent corticomotor excitability.

Several mechanisms could account for the reduced capacity of low-frequency rTMS-induced plasticity observed in the older adults. Changes in neuronal morphology, such as an atrophy of cortical gray matter (Good et al., 2001) and motorneurons (Henderson et al., 1980), declining motor axon conduction velocities (Jankelowitz et al., 2007), and decreasing motor cortical synaptic connectivity (Adams, 1987a,b) have all been reported with increasing age and could play a role in these findings. Additionally, several researchers have suggested an increase in the distance between the stimulating coil and the underlying motor cortex with age may account for the stunted rTMS effects (Kozel et al., 2000; Wagner et al., 2004, 2008). However, in the present study, the older subjects had normal MRI and neurological exams, indicating that the reduced efficacy of TMS we describe is unlikely a consequence of cortical atrophy.

### Reduced hemispheric lateralization in aged subjects

In this study we collected data for single pulse stimulation applied over both hemispheres. This additional data enabled us to investigate the changes in hemispheric asymmetry and inhibitory interactions that occur during the aging process.

Both before and after rTMS intervention, significant differences between young and old subjects were detected only in the right, non-dominant hemisphere. Both before and after rTMS intervention, significant differences between young and old subjects were detected only in the right, non-dominant hemisphere. This result, we believe, provides primary neurophysiological support for the right hemi-aging model that has been proposed to explain age-related changes in lateralization. The right hemi-aging model suggests the right hemisphere is more sensitive aging effects than the left hemisphere (Dolcos et al., 2002; Bernard and Seidler, 2012). It has been suggested that this hemispheric difference is due to a smaller gray-to-white matter ratio in the right than left hemisphere (Gur et al., 1980; Good et al., 2001; Pujol et al., 2002). In addition to the hemi-aging model, another model, termed the Hemispheric Asymmetry Reduction in Older Adults (HAROLD) model has been suggested to account for the differential lateralization patterns observed in young and old subjects. Specifically, the HAROLD model suggests that prefrontal cortical activity tends to be less lateralized in older adults than in younger adults. However, we believe that our present findings are more in accordance with the hemi-aging model rather than the HAROLD model. The HAROLD model, based on neuroimaging evidence, appears to apply to the prefrontal cortex, while the hemi-aging model applies to other brain regions (Cabeza et al., 2002), including the motor cortex, which was the targeted region in the present study.

In the analysis of the MEP data collected after rTMS intervention, we found significant and negative correlations between age and excitability in both hemispheres. A growing body of evidence suggests that lower frequencies of rTMS in the 1 Hz range can suppress excitability of the motor cortex (for review see Kobayashi and Pascual-Leone, 2003). The rTMS applied in this experiment can thus be thought of disrupting the function of the motor cortex, creating a temporary “virtual lesion” (Pascual-Leone, 1999). Based on the post-rTMS correlation analyses, the induction of a virtual lesion in this experiment, on average, increased excitability in the dominant hemisphere. This result is consistent with evidence suggesting that aging is associated with reduced anatomical integrity of the corpus callosum (Abe et al., 2002; Sullivan et al., 2002).

## Conclusions

With regards to our initial inquiry regarding the underlying causes of ipsilateral activation during dominant hand motor movement in older populations, our data appears to support the theory of disrupted inhibitory signal transmission from the dominant M1 (rather than active functional recruitment).

Behaviorally, there were significant increases for both the young and old groups in the RT task in the left (contralateral) hand following rTMS. This result is consistent with the neurophysiologic findings of a greater inter-hemispheric impact of 1 Hz rTMS. It is worth reiterating that our relatively small sample and unbalanced group sizes likely influenced our results. However, we hope these findings inspire additional and larger research projects within this field.

### Conflict of interest statement

The authors declare that the research was conducted in the absence of any commercial or financial relationships that could be construed as a potential conflict of interest.
